# Parallels in the pathogenesis of SARS-CoV-2 and *M. tuberculosis*: a synergistic or antagonistic alliance?

**DOI:** 10.2217/fmb-2020-0179

**Published:** 2021-01-06

**Authors:** Kesego Tapela, Charles Ochieng’ Olwal, Osbourne Quaye

**Affiliations:** ^1^West African Centre for Cell Biology of Infectious Pathogens (WACCBIP), University of Ghana, Accra, Ghana; ^2^Department of Biochemistry, Cell & Molecular Biology, College of Basic & Applied Sciences, University of Ghana, Accra, Ghana

**Keywords:** angiotensin-converting enzyme 2 (ACE2) receptor, BCG vaccine, coronavirus, COVID-19, cytokine storm, lymphocytopenia, *Mycobacterium tuberculosis*, pathogenesis of SARS-CoV-2, SARS-CoV-2, tuberculosis

The world is facing a major challenge of the new pneumonia condition termed COVID-19 caused by SARS-CoV-2, the seventh member of human coronaviruses. The global burden of COVID-19 is rising daily and as of 10 November 2020, there were over 1,275,122 deaths (https://www.worldometers.info/coronavirus/).

Coronaviruses are enveloped, positive-sense and single-stranded RNA viruses [1]. Based on 96.2% nucleotide sequence identity with a bat-borne coronavirus (BatCoV RaTG13) that has been identified in *Rhinolophus affinis* bat species, it is likely that SARS-CoV-2 originated from a bat [2]. COVID-19 is primarily defined by an acute viral pneumonia and cytokine storm leading to respiratory failure [3]. The main transmission route of this virus is droplets blown out through cough and sneezing by an infected person. The common symptoms of COVID-19 include cough, fever, shortness of breath and tiredness [4]. Severe cases manifest in symptoms that are associated with cellular immune deficiency, coagulation activation, myocardia, multiple organ dysfunction and septic shock [5]. SARS-CoV-2 is highly pathogenic in persons with underlying medical conditions that reduce their immune competence such as TB [6].

TB, caused by *Mycobacterium tuberculosis* and transmitted through infected air droplets from cough or sneezing, is one of the top ten causes of death due to infectious diseases globally, with an estimated 10 million infections and 1.5 million deaths in 2018 [7]. Indeed, since TB affects the respiratory system, it could prove catastrophic if present in comorbidity with COVID-19 [6].

In this commentary, we discuss the current state of knowledge on the parallels in the pathogenesis of SARS-CoV-2 and *M. tuberculosis* and the potential implications of co-infection on the clinical outcomes.

## SARS-CoV-2 & *M. tuberculosis*: targeting same cells?

Spike (S) glycoprotein on the surface of SARS-CoV-2 facilitates viral entry into target cells. The S protein consists of three receptor-binding S1 heads which bind to the cellular receptor to facilitate viral attachment to target cells [8]. Moreover, the S protein is required to be proteolytically active by being cleaved at the multibasic S1/S2 cleavage site by the host’s cell protease furin to facilitate protein-mediated cell-cell fusion [9]. The receptor binding domain of the S protein binds to angiotensin-converting enzyme 2 (ACE2) receptor and invade the host cell through clathrin-mediated endocytosis [4]. Type I interferons (IFN) stimulate the expression of SARS-CoV-2 entry receptors and enable the virus to be endocytosed [4]. The high human ACE2 binding affinity of the receptor binding domain and furin activation of the spike helps SARS-CoV-2 to successfully enter the human lung cell while evading the immune response [10]. The virus infects and replicates in ciliated and mucus-secreting cells of bronchial epithelium, type II pneumocytes in the lungs [11] and macrophages [12].

Like SARS-CoV-2, *M. tuberculosis* also infects type II pneumocytes in the lungs [13,14] via pattern recognition receptors of the host cells, including toll-like receptors, complement receptors, dendritic cell-specific intercellular adhesion molecule grabbing nonintegrin (DC-SIGN), mannose receptors, CD14 receptors, scavenger receptors and FC*γ* receptors [15]. Furthermore, the bacterium is internalized through phagocytosis by the resident macrophages and further translocated to lysosomes for destruction [16].

Co-infection of SARS-CoV-2 and *M. tuberculosis* may exacerbate the pathologies that are associated with each pathogen. The co-infection in macrophages can increase the production of pro- and anti-inflammatory cytokines and therefore play a crucial role in pathogenesis. Severe infection of type II pneumocytes with SARS-CoV-2 can cause regeneration failure of the cells and lead to lesions in the lungs, accelerated bronchial damage and ultimately lung disease [17].

## Dysregulating the immune system: the shared story of SARS-CoV-2 & *M. tuberculosis*

The human immune system responds to SARS-CoV-2 by differentiating the monocytes into pro-inflammatory macrophages through activation of JAK–STAT pathways, an activation that is mediated by production of IL-1β and/ or IL-18 [18]. The activation can lead to the production of pro-inflammatory cytokines and resulting in COVID-19-associated cytokine storm. Cytokine storm causes rapid replication of SARS-CoV-2 virus and results in the infection of more alveolar epithelial cells [19]. Significant elevation of inflammatory cytokines such as IL-6, IL-1β and IP10 have been detected in COVID-19 patients [20]. The rapidly increasing cytokines induce excess production of neutrophils, which infiltrate lung tissue and cause lung injury [21]. Cytokine storm is also induced in *M. tuberculosis* infection and is driven by an overwhelming immune response from alveolar macrophages, monocytes and dendritic cells in the lungs and lymph nodes [22]. These cells release an array of cytokines, including IL-1, IL-18, IFN-α, IL-6, IL-10 [23] and therefore co-infections of SARS-CoV-2 with *M. tuberculosis* will likely result in the production of high amounts of cytokines and lead to exacerbated pathogenicity.

Although the two pathogens share some features of cytokine storm, it is important to note that the magnitude of response differs, with *M. tuberculosis* having a lower response. Moreover, the outcomes associated with cytokine storm are different for the two pathogens. The COVID-19 patients experience acute respiratory distress syndrome whereas in *M. tuberculosis* chronic wasting and slow long destruction ensue from cytokine storm.

Another marked feature in the immune response against SARS-CoV-2 is lymphocytopenia [24]. Lymphocytes count of less than 1.5 × 10^9^ per liter has been shown to increase the risk of severe COVID-19 [25]. It is believed that the virus directly infects lymphocytes through the ACE2 receptor and kill them leading to lymphocytopenia [26]. The direct damage of organs such as the lymphatic system by SARS-CoV-2 may also result in a reduction of lymphocytes. Production of TNFα, IL-6 and other pro-inflammatory cytokines during SARS-CoV-2 can also induce lymphocyte apoptosis [26].

The dysregulation of T lymphocytes has also been demonstrated in TB patients [27]. It has been reported that protective immunity to TB depends on CD4^+^ cells through cell-mediated responses to allow control of *M. tuberculosis* infection [28]. A depletion of the CD4^+^ cells increases risks to reactivation of TB [29], and is also associated with severe radiological lesions, increased paradoxical reaction or death [27,30]. The depletion of CD4^+^ by TB may also result in immunosuppression, which may increase severity on patients with comorbidity of TB and SARS-CoV-2 [31].

## Potential protective effects of BCG vaccine against COVID-19

BCG is a live attenuated vaccine that has been used to protect children against TB and other viral infections [32]. The vaccine induces immune response through production of antibodies that prevent the proliferation of the pathogen [33]. It is suggested that BCG vaccine might boost the immune system and help reduce the severity of COVID-19 [34]. BCG vaccination has a nonspecific or heterologous effect, which reduces morbidity and mortality to subsequent infections [35]. The vaccine activates CD4^+^ and CD8^+^ memory cells with nontargeted antigens, thus modulating T helper (Th) 1 and Th17 responses for secondary infections [36]. BCG also induces immunological memory in innate immune cells including natural killer (NK) cells, monocytes and macrophages [37]. All these result in bystander response including enhanced production of pro-inflammatory cytokines such as IL-1β, IFN-γ, TNF and IL-6, macrophage activity, T-cell responses and enhanced antibody titers [35].

Based on the nonspecific nature of BCG and its capacity to elicit both innate and adaptive immunity, BCG vaccine has been suggested as a preventive measure against SARS-CoV-2 infection or to reduce COVID-19 severity, especially among children who have already been vaccinated with BCG [38,39]. Healthy individuals vaccinated with BCG will have a boosted immune system, which upon infection with SARS-CoV-2, will lead to inhibited viral replication, decreased viral loads and consequently less inflammation and reduced possibility of cytokine storm production and lymphopenia [38]. Countries in Africa, Asia and South America, that are implementing mandatory national BCG vaccination for their populations, have demonstrated lower COVID-19 cases and mortality [39,40]. Although, a direct link between the low COVID-19 cases and BCG is yet to be established, it is probable that BCG vaccination induces protection against SARS-CoV-2 via trained innate immunity. BCG vaccination results in epigenetically trained populations of monocytes and/or NK cells, which robustly responds to and clears a broad spectrum of viruses, including SARS-CoV-2 [41]. More scientific evidence is needed to establish the protective nature of BCG to SARS-CoV-2 specifically, and to which group of individuals – children and/or adults – should be targeted to realize the full benefits of this trained immunity. Moreover, considering that heightened immune response such as cytokine response is attributed to complications in COVID-19 patients [42], the potential negative effects of elevated immune response by the BCG’s trained immunity warrants further investigation.

## Conclusion & future perspective

SARS-CoV-2 and *M. tuberculosis* infections have some important parallels with regards to cell entry, immune response and immune evasion. Thus, TB-endemic regions may have exacerbated pathologies in the context of SARS-CoV-2 infections; something that the scientific community will need to pay attention to. At the moment, recommending BCG vaccine for combating COVID-19 must be done with caution. Moreover, the vaccine does not offer a lifetime protection and there is paucity of evidence confirming its protective role against SARS-CoV-2. More research should be directed toward a deeper understanding of how BCG might protect against COVID-19. A thorough understanding of the pathophysiology of COVID-19 in the context of *M. tuberculosis* co-infection is warranted to inform on better ways to manage such cases and avert high COVID-19-related mortalities that are likely to occur in the high TB endemic areas.
